# Humoral and cellular immune responses to the mRNA-1273 SARS-CoV-2 vaccine booster in patients on maintenance dialysis

**DOI:** 10.1007/s40620-022-01371-4

**Published:** 2022-06-22

**Authors:** Hristos Karakizlis, Vipul Agarwal, Mostafa Aly, Kevin Strecker, Benjamin Csala, Isla Esso, Jiangping Chen, Christian Nahrgang, Martin Wolter, Heiko Slanina, Christian G. Schüttler, Sönke Jessen, Claudio Ronco, Werner Seeger, Rolf Weimer, Martina Sester, Horst-Walter Birk, Faeq Husain-Syed

**Affiliations:** 1 Department of Internal Medicine II, University Hospital Giessen and Marburg, Justus-Liebig-University Giessen, Klinikstraße 33, 35392 Giessen, Germany; 2 Transplantation Immunology, Institute of Immunology, University Hospital Heidelberg, Im Neuenheimer Feld 305, 69120 Heidelberg, Germany; 3 Nephrology Unit, Internal Medicine Department, Assiut University, Assiut, Egypt; 4 AID GmbH, Ebinger Straße 4, 72479 Strassberg, Germany; 5 Institute of Medical Virology, Justus-Liebig-University Giessen, Schubertstraße 81, 35392 Giessen, Germany; 6 Department of Nephrology, Dialysis and Transplantation, International Renal Research Institute of Vicenza, San Bortolo Hospital, Via Rodolfi, 37, 36100 Vicenza, Italy; 7 Department of Medicine (DIMED), Università di Padova, Via Giustiniani, 2, 35128 Padua, Italy; 8 Universities of Giessen and Marburg Lung Center (UGMLC), Member of the German Center for Lung Research (DZL), Klinikstraße 33, 35392 Giessen, Germany; 9 Department of Lung Development and Remodeling, Max Planck Institute for Heart and Lung Research, Ludwigstraße 43, 61231 Bad Nauheim, Germany; 10 Department of Transplant and Infection Immunology, Saarland University, Kirrberger Straße, 66421 Homburg, Germany; 11 Division of Nephrology and Kidney Transplantation, Department of Internal Medicine II, University Hospital Giessen and Marburg, Klinikstrasse 33, 35392 Giessen, Germany; 12 Division of Nephrology and Critical Care Medicine, Department of Internal Medicine II, University Hospital Giessen and Marburg, Klinikstrasse 33, 35392 Giessen, Germany

Maintenance dialysis patients have higher coronavirus disease 2019 (COVID-19)-related mortality risk than the general population [1]. We and others have shown that patients have waning early antibody-mediated and blunted T cell-mediated immune responses to severe acute respiratory syndrome coronavirus 2 (SARS-CoV-2) vaccination [1, 2]. Optimizing the vaccination strategy in this population requires an understanding of the humoral and cellular immune response dynamics to SARS-CoV-2 vaccines, but immunogenicity data post-booster after primary COVID-19 vaccine cycle are scarce [3]. Here, we report follow-up data on the immune responses 6 months after primary COVID-19 vaccine cycle (T3) and 4 weeks post-booster (T4) following heterologous and homologous primary COVID-19 vaccine cycle SARS-CoV-2 vaccinations in adult patients receiving thrice weekly, in-center dialysis (hemodialysis and peritoneal dialysis) at the University Hospital Giessen and Marburg, Giessen, Germany [1].

We assessed anti-SARS-CoV-2 spike antibodies using a dot plot array (GenID, Strassberg, Germany) and chemiluminescent microparticle immunoassay (Anti-S AdviseDx anti-SARS-CoV-2 spike antibodies II, Abbott, Chicago, IL, USA), and T-cell responses by interferon (IFN)-γ and interleukin (IL)-2 peripheral blood leukocyte secretion upon SARS-CoV-2 glycoprotein stimulation (ELISpot assay, GenID; Supplementary Methods, Supplementary Table S1: study methods, statistical analysis, patients’ characteristics). The local human research ethics committee (AZ 126/21) approved this study and it complied with the Declaration of Helsinki tenets. All participants provided written informed consent before study enrollment.

Of the original cohort (*n* = 60), 47 patients (78.3%) were available for follow-up (T3: *n* = 42; T4: *n* = 46; five patients were transferred to other dialysis centers; six patients died from non-COVID-19-associated causes; two patients received boosters outside their dialysis center). Two patients had asymptomatic COVID-19 breakthrough infection despite complete primary COVID-19 vaccine cycle and therefore were only tested at T4 (Supplementary Table S2). The results of the timepoints T1–T2 around the primary COVID-19 vaccine cycle were recently published [1].

All patients received the mRNA-1273 mRNA-based vaccine booster (Moderna Biotech). Figure 1 depicts the humoral and cellular response dynamics 6 weeks (T2), and 6 months (T3) after primary COVID-19 vaccine cycle and 4 weeks (T4) after booster vaccination. The median anti-SARS-CoV-2 spike antibody levels (Abbott array) were significantly lower at T3 than T2 (501 [interquartile range, 134–1703] vs. 2240 [756–7687] arbitrary units [AU]/ml; *P* < 0.001), increasing markedly to 40,000 [6855–40,000] AU/ml post-booster (*P* < 0.001; Supplementary Tables S3, S4). No changes were observed for percent positivity status across T1–T4 (Fig. 1C).

**Fig. 1:**
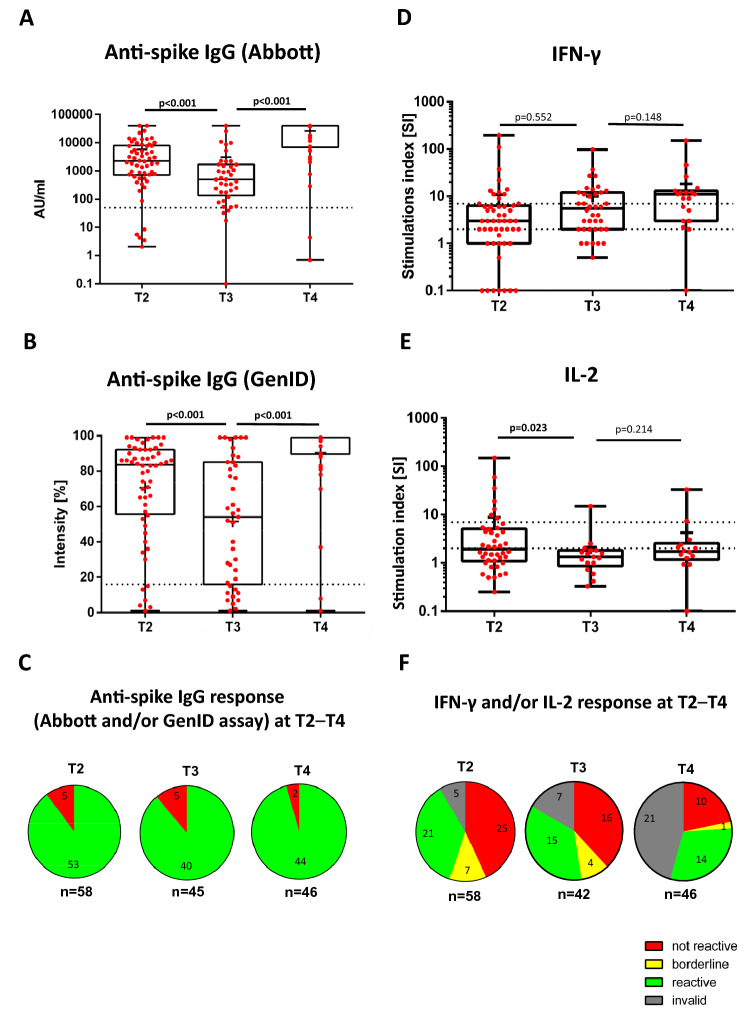
Vaccine-induced anti-SARS-CoV-2 spike antibody detected using the Abbott array (**A**), GenID assay (**B**), and/or both (**C**), and SARS-CoV-2-specific T cell responses with secretion of IFN-γ (**D**), IL-2 (**E**), and/or both (**F**) at T2–T4. The figure depicts the cellular and humoral responses at 6 weeks (T2), 6 months (T3) after basic vaccination, and at 4 weeks (T4) post-booster. The humoral response level (as determined by the Abbott array and GenID assay) was lower at T3 compared to T2 (*P* < 0.001) but increased post-booster (*P* < 0.001). There was no reduction in the IFN-γ response between T2 to T3 (*P* = 0.552) while the SARS-CoV-2-specific IL-2 response was reduced between both timepoints (*P* = 0.023). No increase in cellular response (IL-2 or IFN-γ) was observed post-booster (p = NS). A logarithmic scale was used on the y-axis in panel A, D, and E. Due to the log scale, anti-SARS-CoV-2 spike antibody (Abbott array), IFN-γ, and IL-2 levels of zero are not displayed. The dashed horizontal lines indicate the cut-off for positivity (reactive; i.e., IgG > 50 AU/ml [Abbott array] and > 16% [GenID assay], IFN-γ and IL-2: SI ≥ 7); the area between the horizontal lines indicates the borderline zone used in each GenID assay. Bold values denote statistical significance at the *P* < 0.05 level. *AU* arbitrary unit, *IFN-γ* interferon-γ, *IgG* immunoglobulin G, *IL-2* interleukin-2, *SARS-CoV-2* severe acute respiratory syndrome coronavirus type 2, *NS* not significant, *T2* timepoint 2, *T3* timepoint 3, *T4* timepoint 4

The median IL-2 stimulation index levels were lower at T3 than T2 (*P* = 0.023) but not the IFN-γ stimulation index levels (*P* = 0.552) between both timepoints (Fig. 1D–E, Supplementary Table S3). Notably, IFN-γ stimulation index levels were higher at T4 than T2. No changes were observed when comparing the percent reactive pattern of the IFN-γ and/or IL-2 ELISpot assays across T1–T4, but the results were flawed due to the high number of invalid samples (Fig. 1F).

The GenID assay demonstrated that patients with IFN-γ-producing T cells had higher anti-SARS-CoV-2 spike antibody levels at T3 (*P* = 0.028, *n* = 30) but not the Abbott array (*P* = 0.08; *n* = 28). At T4, there was no significant difference for either assay (Abbott array: *P* = 0.51, *n* = 17; GenID assay: *P* = 0.442, *n* = 17). IL-2 could not be analyzed due to the low numbers on the reactive side at T3 (*n* = 1) and T4 (*n* = 3).

Patients with COVID-19 history had sustained higher anti-SARS-CoV-2 spike antibody levels (Abbott array) compared to infection-naïve patients at T2 (*n* = 5 vs. 53, respectively, total number = 58) (*P* < 0.001) and T3 (*n* = 5 vs. 35, respectively, total number = 40) (*P* = 0.002; Supplementary Table S5), although the booster conferred median IgG levels reaching the upper detection limit of 40,000 AU/ml in both groups at T4 (*n* = 6 vs. 36, respectively, total number = 42). Patients with COVID-19 history also had higher SARS-CoV-2-specific IFN-γ levels at T2 (*P* < 0.001), but not IL-2 (*P* = 0.07). No differences were seen in the IFN-γ SI levels at T3 (*P* = 0.252) and T4 (*P* = 0.299) between both groups (Supplementary Table S6). Given the high number of invalid samples of patients with COVID-19 history, the T3 and T4 IL-2 immune responses could not be analyzed.

 Our results indicate a robust humoral immune response 6 months following primary COVID-19 vaccine cycle (> 90%), which is consistent with previous reports involving hemodialysis patients and healthy controls [3, 4]. However, while primary COVID-19 vaccine cycle resulted in markedly high anti-SARS-CoV-2 spike antibody levels (levels were highest in patients with previous COVID-19), the humoral response waned significantly within 6 months. IgG seropositivity, defined by commercially available tests, may overestimate the effectiveness of vaccine-induced humoral immunity, as the cutoff value that correlates with protection against SARS-CoV-2 infection is unknown. In contrast, we observed a sustained weak cellular immune response post-booster, although IFN-γ stimulation index levels increased significantly. Therefore, in line with previous works [4], antibody presence may not automatically correlate with functional cellular immunity, which is likely an important component in long-term protection against SARS-CoV-2. We and others have previously shown that cytokine induction during primary infection is associated with preferential induction of T cells producing IL-2, whereas reactivations are associated with T cells producing IFN [5]. This may also be applicable to booster vaccinations, as shown in the present study. Overall, our data indicate progressive waning of humoral immunity and a sustained weak cellular immune response within 6 months; the booster vaccination is able to substantially increase humoral immunity again; the emergence of SARS-CoV-2 variants with high potential for immune evasion may necessitate a further booster dose 4–6 months after the previous booster vaccination in dialysis patients.

## Supplementary Information

Below is the link to the electronic supplementary material.

Supplementary file1 (DOCX 82 KB)

## Declarations

### Availability of data and material

The data that support the findings of this study are available from the corresponding authors upon reasonable request.
