# Supplementary material for: Humoral and cellular immune responses to the mRNA-1273 SARS-CoV-2 vaccine booster in patients on maintenance dialysis
Source: J Nephrol. 2022 Jun 22;36(1):183–6. doi: 10.1007/s40620-022-01371-4 (PMC9214671; doi:10.1007/s40620-022-01371-4)
Supplement: Supplementary file 1 — Supplementary file1 (DOCX 82 KB) [file 40620_2022_1371_MOESM1_ESM.docx]

SUPPLEMENTARY MATERIAL

**Humoral and cellular immune responses to the mRNA-1273 SARS-CoV-2 vaccine booster in patients on maintenance dialysis**

Hristos Karakizlis MD, Vipul Agarwal, Mostafa Aly MD, Kevin Strecker, Benjamin Csala, Isla Esso, Jiangping Chen PhD, Christian Nahrgang, Martin Wolter, Heiko Slanina MD, Christian G. Schüttler MD, Sönke Jessen MD, Claudio Ronco MD, Werner Seeger MD, Rolf Weimer MD, Martina Sester PhD, Horst-Walter Birk MD, Faeq Husain-Syed MD

This supplementary material has been provided by the authors to give readers additional information about their work.

TABLE OF CONTENTS

[SUPPLEMENTAL METHODS 3](#_Toc104738348)

[*Study design and participants* 3](#_Toc104738349)

[ELISpot Assay 3](#_Toc104738350)

[*Analysis of SARS-CoV-2-specific antibodies* 3](#_Toc104738351)

[Other laboratory methods 3](#_Toc104738352)

[Other measures 3](#_Toc104738353)

[Statistical analysis 4](#_Toc104738354)

[ONLINE TABLES 5](#_Toc104738355)

[*Table S1:* Demographic and clinical characteristics of the follow-up cohort 5](#_Toc104738356)

[*Table S2*: Clinical characteristics of patients with COVID-19 breakthrough infection despite complete prime-boost vaccination 7](#_Toc104738357)

[*Table S3:* Cellular and humoral immune responses at 6 weeks (T2) after SARS-CoV-2 primary COVID-19 vaccine cycle and 1 week (T3) before booster vaccination 8](#_Toc104738358)

[*Table S4:* Cellular and humoral immune responses 6 months after primary COVID-19 vaccine cycle (T3) and 4 weeks after (T4) SARS-CoV-2 booster vaccination 9](#_Toc104738359)

[*Table S5:* Cellular and humoral immune responses following SARS-CoV-2 vaccination at T2 and T3 with history or no history of COVID-19 10](#_Toc104738360)

[*Table S6:* Cellular and humoral immune responses months after primary COVID-19 vaccine cycle (T3) and 4 weeks after (T4) SARS-CoV-2 booster vaccination with history or no history of COVID-19 11](#_Toc104738361)

[SUPPLEMENTARY REFERENCES 12](#_Toc104738362)

# SUPPLEMENTAL METHODS

## *Study design and participants*

The study protocol has been described in detail elsewhere [1]. Briefly, patients aged ≥18 years receiving thrice-weekly in-center dialysis (hemodialysis or peritoneal dialysis) were enrolled between March 2021 and June 2021 at the Patienten-Heimversorgung (PHV) outpatient dialysis center at University Hospital Giessen and Marburg, Giessen, Germany. At the time of enrollment, the dialysis center served 84 hemodialysis patients and 5 peritoneal dialysis patients. Patients were enrolled if they had: i) received a homologous mRNA-based or a single-dose or homologous dual dose vector-based vaccine regimen (with or without history of COVID-19), and ii) no laboratory evidence of current SARS-CoV-2 infection. The interval between the first and second injections was determined as per EMA guidelines [2]: 3–4 weeks for homologous mRNA-based vaccines and 4–12 weeks for the vector-based single dose (Ad26.COV2.S) or homologous vector-based ChAdOx1 nCoV-19 vaccine. The interval between the second and third injections was determined as per EMA guidelines: at least 6 months after the second dose [3]. All blood samples related to the follow-up study were obtained prior to dialysis treatment at 6 months after primary COVID-19 vaccine cycle (T3) and 4 weeks after booster vaccination (T4), with a tolerance range of ±2 days.

### ELISpot Assay

A detailed description of the collection and management of serum and peripheral blood mononuclear cell (PBMC) samples is depicted in our previous work [1]. Briefly, SARS-CoV-2-reactive T cell responses were quantified from citrated whole blood using a multicolor FluoroSpot Immune assay kit (AID® GmbH, Straßberg, Germany), which detects functional T cells and an interferon (IFN)-γ and interleukin (IL)-2 reaction specifically against SARS-CoV-2. The results were evaluated by calculating the ratio of the antigen specific reaction and negative control (NC) (stimulation index, SI). Any antigen-specific FluoroSpot test with an SI of ≤2 (NC > 2) or ≤5 (NC < 2), depending on the background stimulation, was considered negative when assessed quantitatively. For NC <2 a stimulation index of > 5 – < 7 was considered borderline and a SI ≥ 7 was considered positive, whereas for NC ≥2 a stimulation index of > 2 – ≤ 3 was considered borderline and a SI > 3 was considered positive. A reactive response was considered when at least one timepoint showed a reactive pattern and the other timepoint was reactive or borderline or negative or invalid. A borderline response was considered when one timepoint was borderline and the other timepoint was borderline or negative or invalid. A negative response was considered when one timepoint was negative and the other timepoint was negative or invalid. Only when both timepoints were invalid was the response considered as invalid. GenID performed all FluoroSpot tests, blinded to the clinical data.

## *Analysis of SARS-CoV-2-specific antibodies*

SARS-CoV-2-specific antibodies were quantified using plasma from citrated whole blood samples using an immunoglobulin G (IgG) assay coated with a recombinant receptor-binding domain of the SARS-CoV-2 spike protein antigen using an in-house dot plot array by GenID, blinded to the clinical data. Antibody levels are expressed in % intensity of gray scale, ranging from 0 to 100 percent black, with an intensity of >16% considered positive and ≤16% considered negative, respectively. In addition, SARS-CoV-2-specific antibodies from serum samples against the spike protein and nucleocapsid protein were performed by the Institute of Medical Virology (Giessen, Germany) using antibody chemiluminescent microparticle immunoassay (CMIA; Anti-S AdviseDx SARS-CoV-2 IgG II and Anti-N Abbott Architect anti-SARS-CoV-2 spike, Abbott, Chicago, IL, USA). Anti-S levels after infection or vaccination were expressed as AU (arbitrary unit)/ml with levels >50AU/ml defined as positive and ≤50 AU/ml as negative.

### Other laboratory methods

All other blood samples were analyzed at the local Institute of Laboratory Medicine at University Hospital Giessen and Marburg, Giessen, where they were processed within 30 min of collection and centrifuged for 10 min at 3000 ×*g*. Serum creatinine was measured by a photometric-enzymatic method on an ADVIA Chemistry XPT analyzer (enzymatic creatinine; Siemens Healthineers, Erlangen, Germany), with calibration to reference measurements for isotope dilution mass spectrometry. Ferritin was measured by chemiluminescent immunoassay (CLIA) on a Centaur XPT analyzer (Siemens Healthineers). Soluble IL-2 receptor and IL-6 were determined on a Siemens Immulite 1000 system with Siemens reagents.

### Other measures

The other variables included kidney failure etiology, dialysis vintage and dose (Kt/V for thrice-weekly hemodialysis [4] and weekly Kt/V for peritoneal dialysis [5]), previous SARS-CoV-2 infection, and use of immunosuppressive therapy.

### Statistical analysis

Descriptive statistics are expressed as the median [interquartile range] for numeric variables and as n (%) for categorical variables. Differences between two independent groups were tested with the Mann-Whitney test or independent t-test according to the variables’ distribution normality. Categorical variables were tested with the chi-square test or Fisher’s exact test. Paired ordinal data were compared using the Wilcoxon signed-rank test; paired nominal data were compared using the McNemar test. The statistical analysis was performed with SPSS Statistics 26 (IBM, Ehningen, Germany). P-values <0.05 were considered significant.

# ONLINE TABLES

## *Table S1:* Demographic and clinical characteristics of the follow-up cohort

|  | **Total**  **(n = 47)** | **mRNA-based primary COVID-19 vaccine cycle (mRNA-1273/BNT162b2)**  **(n = 41)** | **Vector-based primary COVID-19 vaccine cycle (ChAdOx1 nCoV-19Ad26.COV2-S)**  **(n = 6)** | **p-value** |
| --- | --- | --- | --- | --- |
| **Demographics** |  |  |  |  |
| Age, years | 67 [56–77] | 65 [52–77] | 72 [64–76] | 0.37 |
| Male sex, n (%) | 26 (61.9%) | 28 (68.3%) | 3 (50.0%) | 0.38 |
| Dry weight, kg | 79.7 [69.9–89.6] | 77.3 [69.6–89.9] | 84.8 [75.3–91.1] | 0.67 |
| Body mass index, kg/m^2^ | 26.8 [23.1–30.4] | 26.6 [23.0–30.1] | 28.4 [25.3–33.7] | 0.43 |
| **Comorbidities** |  |  |  |  |
| Hypertension, n (%) | 35 (74.5%) | 31 (75.6%) | 4 (66.7%) | 0.64 |
| Diabetes mellitus, n (%) | 15 (31.9%) | 13 (31.7%) | 2 (33.3%) | 0.94 |
| Coronary artery disease, n (%) | 20 (42.6%) | 18 (43.9%) | 2 (33.3%) | 0.63 |
| History of stroke, n (%) | 7 (14.9%) | 6 (14.6%) | 1 (16.7%) | 0.90 |
| Immunosuppressive therapy, n (%) | 7 (14.9%) | 6 (14.6%) | 1 (16.7%) | 0.35 |
| Cause of kidney failure, n (%) |  |  |  | 0.75 |
| *Nephrosclerosis* | 10 (21.3%) | 9 (22.0%) | 1 (16.7%) |  |
| *Diabetic nephropathy*  */nephrosclerosis* | 9 (19.1%) | 7 (17.1%) | 2 (33.3%) |  |
| *Cardiorenal syndrome* | 7 (14.9%) | 7 (17.1%) | 0 (0%) |  |
| *Glomerulonephritis* | 9 (19.1%) | 7 (17.1%) | 2 (33.3%) |  |
| *Interstitial nephritis* | 3 (6.4%) | 3 (7.3%) | – |  |
| *ADPKD* | 3 (6.4%) | 3 (7.3%) | – |  |
| *Cancer* | 4 (8.5%) | 3 (7.3%) | 1 (16.7%) |  |
| *Unknown* | 2 (4.3%) | 2 (4.9%) | – |  |
| **Dialysis data** |  |  |  |  |
| Dialysis modality, n (%) |  |  |  | 0.42 |
| *Hemodialysis* | 43 (88.3%) | 37 (90.2%) | 6 (100%) | ~~0.69~~ |
| *Peritoneal dialysis* | 4 (11.7%) | 4 (9.8%) | – |  |
| Dialysis vintage, months | 32.5 [15.8–47.5] | 33.3 [15.3–48.5] | 27.5 [16.0–63.0] | 0.71 |
| Kt/V | 1.6 [1.4–1.7] | 1.6 [1.4–1.7] | 1.4 [1.3–1.6] | 0.17 |
| *Hemodialysis* | 1.5 [1.4–1.7] | 1.6 [1.4–1.7] | 1.4 [1.3–1.5] |  |
| *Peritoneal dialysis* | 2.5 [1.8–4.8] | 2.5 [1.8–4.8] | – |  |
| **Baseline clinical data** |  |  |  |  |
| Leukocyte count, g/l | 6.5 [5.1–7.8] | 6.5 [5.1–8.1] | 5.9 [5.1–6.6] | 0.30 |
| Differential count, g/l |  |  |  |  |
| Total neutrophils | 4.5 [3.3–5.6] | 4.7 [3.2–5.7] | 3.8 [3.5–4.4] | 0.44 |
| Total lymphocytes | 1.1 [0.9–1.4] | 1.1 [0.9–1.4] | 1.2 [0.8–1.3] | 0.99 |
| Total basophils | 0.04 [0.03–0.06] | 0.04 [0.03–0.06] | 0.06 [0.03–0.06] | 0.72 |
| Total monocytes | 0.7 [0.5–0.8] | 0.7 [0.5–0.8] | 0.6 [0.5–0.8] | 0.64 |
| Total eosinophils | 0.2 [0.1–0.3] | 0.2 [0.1–0.3] | 0.2 [0.04–0.3] | 0.58 |
| Hemoglobin, g/dl | 10.6 [10.2–11.2] | 10.6 [10.2–11.2] | 10.7 [9.8–11.6] | 0.88 |
| Serum creatinine, mg/dl^a^ | 7.6 [5.9–9.1] | 7.4 [5.8–8.7] | 9.1 [6.3–9.4] | 0.28 |
| Urea, mg/dl^b^ | 119 [102–143] | 120 [102–145] | 109 [78–143] | 0.35 |
| Phosphate, mmol/l | 1.8 [1.3–2.1] | 1.8 [1.3–2.1] | 1.7 [1.3–2.0] | 0.79 |
| Parathyroid hormone, pg/ml | 316 [227–453] | 316 [217–462] | 332 [228–401] | 0.85 |
| Albumin, g/dl | 40.8 [38.7–42.4] | 40.8 [38.7–42.3] | 40.9 [38.5–44.3] | 0.47 |
| C-reactive protein, mg/l | 8.5 [3.3–15.9] | 10.3 [4.1–19.2] | 4.8 [2.3–7.5] | 0.10 |
| Total IgG, g/l | 11.0 [8.4–14.2] | 11.9 [8.3–14.6] | 10.4 [7.7–11.7] | 0.35 |
| IL-6, µg/l | 10.0 [10.0–10.5] | 10.0 [10.0–10.0] | 10.0 [10.0–13.8] | 0.99 |
| Soluble IL-2 receptor, U/ml | 1145 [872–1560] | 1145 [891–1495] | 1301 [716–1755] | 0.99 |
| Ferritin, µg/l | 209 [72–309] | 235 [69–312] | 165 [114–318] | 0.79 |

Values are the median [interquartile range] or n (%).

ADPKD, autosomal dominant polycystic kidney disease; IgG, immunoglobulin G; IL-2, interleukin-2; IL-6, interleukin-6.

^a^To convert the values for serum creatinine to µmol/L, multiply by 88.4

^b^To convert the values for urea to BUN, multiply by 0.467.

## *Table S2*: Clinical characteristics of patients with COVID-19 breakthrough infection despite complete prime-boost vaccination

| **Pt** | **Age, yr** | **Sex** | **Vaccine** | **Cause of kidney failure** | **Dialysis vintage, months** | **BMI, kg/m^2^** | **Comorbidities** | **Immunosuppression** | **SARS-CoV-2 anti-spike IgG at T1/T2/T4, AU/ml** | **Vaccine cellular response** |
| --- | --- | --- | --- | --- | --- | --- | --- | --- | --- | --- |
| 1 | 73 | M | BNT162b2 (Pfizer/BioNTech) | Glomerulonephritis | 17 | 26.5 | Hypertension | None | 2575/1849/40,000 | Negative at T1/T2/T4 |
| 2 | 70 | M | BNT162b2 (Pfizer/BioNTech) | Nephrosclerosis | 26 | 18.4 | Hypertension | None | 313/396/3108 | Positive at T2/T4 |

Please note that no data are available at timepoint T3 for Pts 1 and 2.

AU, arbitrary unit; BMI, body mass index; COVID-19, coronavirus disease 2019; IgG, immunoglobulin G; SARS-CoV-2, severe acute respiratory syndrome coronavirus type 2; T1, timepoint 1; T2, timepoint 2; T4, timepoint 4.

## *Table S3:* Cellular and humoral immune responses at 6 weeks (T2) after SARS-CoV-2 primary COVID-19 vaccine cycle and 1 week (T3) before booster vaccination

|  | **Vaccines (mRNA- and vector-based)** | | **p-value** |
| --- | --- | --- | --- |
|  | T2 | T3 |  |
| anti-SARS-CoV-2 spike antibody, AU/mL (Abbott array) | 2240 [756–7687]  (n = 48) | 500.7 [134–1703]  (n = 40) | **<0.001** |
| anti-SARS-CoV-2 spike antibody% (GenID assay) | 84.5 [58–92.7]  (n = 48) | 53 [15.5–85]  (n = 40) | **<0.001** |
| Interleukin-2, SI | 1.6 [1.0–4.7]  (n = 33) | 1.4 [1.0–1.8]  (n = 16) | **0.023** |
| Interferon-γ, SI | 3 [1–7]  (n = 43) | 6 [2–12]  (n = 34) | 0.552 |

Values are the median [interquartile range]. Bold values denote statistical significance at the p <0.05 level.

AU, arbitrary unit; IgG, immunoglobulin G; SARS-CoV-2, severe acute respiratory syndrome coronavirus type 2; T2, timepoint 2; T3, timepoint 3.

## *Table S4:* Cellular and humoral immune responses 6 months after primary COVID-19 vaccine cycle (T3) and 4 weeks after (T4) SARS-CoV-2 booster vaccination

|  | **Vaccines (mRNA- and vector-based)** | | **p-value** |
| --- | --- | --- | --- |
|  | T3 | T4 |  |
| anti-SARS-CoV-2 spike antibodies ~~,~~ AU/mL (Abbott array) | 500.7 [134–1703]  (n = 40) | 40,000 [6855–40,000]  (n = 42) | **<0.001** |
| anti-SARS-CoV-2 spike antibodies, % (GenID assay) | 53 [15.5–85]  (n = 40) | 99 [89–99]  (n = 44) | **<0.001** |
| Interleukin-2, SI | 1.4 [1.0–1.8]  (n = 16) | 1.8 [1.3–2.6]  (n = 13) | 0.214 |
| Interferon-γ, SI | 6 [2–12]  (n = 34) | 11 [3–13]  (n = 19) | 0.148 |

Values are the median [interquartile range]. Bold values denote statistical significance at the p <0.05 level.

AU, arbitrary unit; IgG, immunoglobulin G; SARS-CoV-2, severe acute respiratory syndrome coronavirus type 2; T3, timepoint 3; T4, timepoint 4.

## *Table S5:* Cellular and humoral immune responses following SARS-CoV-2 vaccination at T2 and T3 with history or no history of COVID-19

|  | **Vaccinated with no history of COVID-19** | | **Vaccinated with history of COVID-19** | | **p-value** |
| --- | --- | --- | --- | --- | --- |
|  | **Median [IQR]** | **n** | **Median [IQR]** | **n** |  |
| anti-SARS-CoV-2 spike antibodies at T2, AU/ml (Abbott) | 1894 [583–5268] | 53 | 14,653 [10584–40,000] | 5 | **<0.001** |
| anti-SARS-CoV-2 spike antibodies at T3, AU/ml (Abbott) | 362 [87–1422] | 34 | 10,738 [2905–32,749] | 6 | **0.002** |
| anti-SARS-CoV-2 spike antibodies at T2, % (GenID) | 83 [54–91] | 53 | 92 [68.5–97] | 5 | 0.15 |
| anti-SARS-CoV-2 spike antibodies at T3, % (GenID) | 40 [13–81] | 35 | 99 [76.5–99] | 5 | **0.002** |
| Interleukin-2 at T2, SI | 1.67 [1–4.4] | 37 | 35.3 [1.5–105] | 5 | 0.07 |
| Interleukin-2 at T3, SI | 1.4 [1.0–1.8] | 14 | NA | 2 |  |
| Interferon-γ at T2, SI | 3 [1–6] | 47 | 110 [22–186] | 5 | **<0.001** |
| Interferon-γ at T3, SI | 6 [2–12] | 29 | 7 [3.5–67] | 5 | 0.252 |

Values are the median [interquartile range]. Bold values denote statistical significance at the p <0.05 level. Patients with COVID-19 infection after complete basic vaccination are included in the cohort „Vaccinated with history of COVID-19".

AU, arbitrary unit; COVID-19, coronavirus disease 2019; IgG, immunoglobulin G; SARS-CoV-2, severe acute respiratory syndrome coronavirus type 2; T2, timepoint 2; T3, timepoint 3.

## *Table S6:* Cellular and humoral immune responses 6 months after primary COVID-19 vaccine cycle (T3) and 4 weeks after (T4) SARS-CoV-2 booster vaccination with history or no history of COVID-19

|  | **Vaccinated with no history of COVID-19** | | **Vaccinated with history of COVID-19** | | **p-value** |
| --- | --- | --- | --- | --- | --- |
|  | **Median [IQR]** | **n** | **Median [IQR]** | **n** |  |
| anti-SARS-CoV-2 spike antibodies at T3, AU/ml (Abbott) | 362 [87–1422] | 34 | 10,738 [2905–32,749] | 6 | **0.006** |
| anti-SARS-CoV-2 spike antibodies at T4, AU/ml (Abbott) | 40,000 [5418–40,000] | 36 | 40,000 [23,129–40,000] | 6 | 0.493 |
| anti-SARS-CoV-2 spike antibody at T3, % (GenID) | 40 [13–81] | 35 | 99 [76.5–99] | 5 | **0.002** |
| anti-SARS-CoV-2 spike antibody at T4, % (GenID) | 99 [89–99] | 37 | 99 [99–99] | 7 | 0.254 |
| Interleukin-2 at T3, SI | 1.4 [1.0–1.8] | 14 | NA | 2 |  |
| Interleukin-2 at T4, SI | 1.7 [1.2–2.7] | 12 | NA | 1 |  |
| Interferon-γ at T3, SI | 6 [2–12] | 29 | 7 [3.5–67] | 5 | 0.252 |
| Interferon-γ at T4, SI | 9 [3–12] | 14 | 12 [5.5–99] | 5 | 0.298 |

Values are the median [interquartile range]. Bold values denote statistical significance at the p <0.05 level.

AU, arbitrary unit; COVID-19, coronavirus disease 2019; IgG, immunoglobulin G; SARS-CoV-2, severe acute respiratory syndrome coronavirus type 2; T3, timepoint 3; T4, timepoint 4.

SUPPLEMENTARY REFERENCES

1. Karakizlis H, Nahrgang C, Strecker K*, et al.* Immunogenicity and reactogenicity of homologous mRNA-based and vector-based SARS-CoV-2 vaccine regimens in patients receiving maintenance dialysis. Clin Immunol 2022;236:108961

2. European Medicines Agency. COVID-19 vaccines. Accessed July 25, 2021. <https://www.ema.europa.eu/en/human-regulatory/overview/public-health-threats/coronavirus-disease-covid-19/treatments-vaccines/covid-19-vaccines>.

3. European Medicines Agency. COVID-19 vaccines. Accessed March 13, 2022. <https://www.ema.europa.eu/en/news/comirnaty-spikevax-ema-recommendations-extra-doses-boosters>.

4. National Kidney F. KDOQI Clinical Practice Guideline for Hemodialysis Adequacy: 2015 update. Am J Kidney Dis 2015;66(5):884-930

5. Blake PG, Bargman JM, Brimble KS*, et al.* Clinical Practice Guidelines and Recommendations on Peritoneal Dialysis Adequacy 2011. Perit Dial Int 2011;31(2):218-239
